# A key enzyme of animal steroidogenesis can function in plants enhancing their immunity and accelerating the processes of growth and development

**DOI:** 10.1186/s12870-017-1123-2

**Published:** 2017-11-14

**Authors:** George V. Shpakovski, Svetlana G. Spivak, Irina N. Berdichevets, Olga G. Babak, Svetlana V. Kubrak, Alexander V. Kilchevsky, Andrey V. Aralov, Ivan Yu. Slovokhotov, Dmitry G. Shpakovski, Ekaterina N. Baranova, Marat R. Khaliluev, Elena K. Shematorova

**Affiliations:** 1 Shemyakin-Ovchinnikov Institute of Bioorganic Chemistry, Russian Academy of Sciences, Moscow, Russia; 2 Institute of Genetics and Cytology, National Academy of Sciences of Belarus, Minsk, Belarus; 3 Belarusian State Medical University, Minsk, Belarus; 4 Timiryazev Institute of Plant Physiology, Russian Academy of Sciences, Moscow, Russia; 5 All-Russia Research Institute of Agricultural Biotechnology, Moscow, Russia; 6 Russian State Agrarian University – Moscow Timiryazev Agricultural Academy, Moscow, Russia

**Keywords:** Transgenic plants, Cytochrome P450_scc_ (CYP11A1), Tobacco, *Botrytis cinerea*, Progesterone, Mitochondria, Tomato, Abiotic stresses, Steroid hormones

## Abstract

**Background:**

The initial stage of the biosynthesis of steroid hormones in animals occurs in the mitochondria of steroidogenic tissues, where cytochrome P450_SCC_ (CYP11A1) encoded by the *CYP11A1* gene catalyzes the conversion of cholesterol into pregnenolone – the general precursor of all the steroid hormones, starting with progesterone. This stage is missing in plants where mitochondrial cytochromes P450 (the mito CYP clan) have not been found. Generating transgenic plants with a mitochondrial type P450 from animals would offer an interesting option to verify whether plant mitochondria could serve as another site of P450 monooxygenase reaction for the steroid hormones biosynthesis.

**Results:**

For a more detailed comparison of steroidogenic systems of Plantae and Animalia, we have created and studied transgenic tobacco and tomato plants efficiently expressing mammalian *CYP11A1* cDNA. The detailed phenotypic characterization of plants obtained has shown that through four generations studied, the transgenic tobacco plants have reduced a period of vegetative development (early flowering and maturation of bolls), enlarged biomass and increased productivity (quantity and quality of seeds) as compared to the only empty-vector containing or wild type plants. Moreover, the *CYP11A1* transgenic plants show resistance to such fungal pathogen as *Botrytis cinerea*. Similar valuable phenotypes (the accelerated course of ontogenesis and/or stress resistance) are also visible in two clearly distinct transgenic tomato lines expressing *CYP11A1* cDNA: one line (No. 4) has an accelerated rate of vegetative development, while the other (No. 7) has enhanced immunity to abiotic and biotic stresses. The progesterone level in transgenic tobacco and tomato leaves is 3–5 times higher than in the control plants of the wild type.

**Conclusions:**

For the first time, we could show the compatibility in vivo of even the most specific components of the systems of biosynthesis of steroid hormones in Plantae and Animalia. The hypothesis is proposed and substantiated that the formation of the above-noted special phenotypes of transgenic plants expressing mammalian *CYP11A1* cDNA is due to the increased biosynthesis of progesterone that can be considered as a very ancient bioregulator of plant cells and the first real hormone common to plants and animals.

**Electronic supplementary material:**

The online version of this article (10.1186/s12870-017-1123-2) contains supplementary material, which is available to authorized users.

## Background

Sterols, their secondary metabolites and steroid hormones play a significant role in the regulation of several crucial physiological processes in living organisms. Until recently, it was generally accepted that brassinosteroids, the specific class of steroid hormones, play the most important regulatory and signalling roles in plants [1–5]. However, in recent years a significant amount of experimental data have been obtained to suggest that the plant sterols themselves (β-sitosterol, stigmasterol, campesterol and cholesterol) hold considerable regulatory activity, regardless of their conversion to brassinosteroids [6, 7]. In addition, sterols are the primary substrates for the synthesis of a number of secondary metabolites (such as progesterone), which participate in the regulation of physiological functions in plants [8–10]. It also became clear that the most important role in controlling the synthesis of steroid compounds not only in animals, but also in plants is played by such steroidogenic proteins as cytochrome P450 dependent monooxygenases, steroid reductases and ketosteroid isomerases [11–17]. Moreover, some stages of the biosynthesis of brassinosteroids and animal steroid hormones are evolutionarily conserved. For instance, it has been shown that the steroid 5α-reductases of plants (DET2) and animals (5αR) are orthologs and catalyze the same type of reaction in the biosynthesis of steroids: the conversion of progesterone to 5α-pregnane-3,20-dione (5α-dihydroprogesterone, 5α-DHP) in animal cells and the convertion of campestenone into 5α-campestanone – an intermediate in the biosynthesis of plant brassinosteroids [17–19]. The Δ5-hydroxysteroid dehydrogenase/Δ5-Δ4-ketosteroid isomerase (3β-HSD) that plays an important role in the biosynthesis of animal steroid hormones is also found in plants, where it performs the same function [13, 20].

Despite the described similarities of the processes of steroidogenesis in plants and animals, there is at least one fundamental difference between them. The initial stage of animal steroid hormones biosynthesis occurs in the mitochondria of steroidogenic tissues, where cytochrome P450_scc_ encoded by the *CYP11A1* gene with participation of electron transfer chain proteins adrenodoxin reductase [AdR] and adrenodoxin [Ad] catalyzes the conversion of cholesterol into pregnenolone – the general precursor of all steroid hormones, starting with progesterone. Although the existence of homologous to AdR and Ad plant ferredoxin reductase [MFDR] and two closely related ferredoxins [MFDXs] of the mitochondrial type was clearly demonstrated for *Arabidopsis thaliana* [21–23] and recently for some *Digitalis* and *Solanaceae* family species [24], this stage is missing in plants where mitochondrial cytochromes P450 (the mito CYP clan) have not been found. Instead, the biosynthetic pathway for campesterol provides the precursors for brassinosteroids and phytohormones involved in the regulation of plant growth and development. At the same time, a number of animal steroid hormones (pregnenolone sulfate, progesterone, 17-hydroxyprogesterone, 16-dehydroprogesterone, androstenedione) and receptors, which mediate mitochondrial cholesterol uptake in animal cells have also been recently discovered in many divergent plant species (see Additional File 1) [9, 25–27].

In this regard, the development and study of transgenic plants expressing cDNA of a mammalian *CYP11A1* gene encoding for mitochondrial cytochrome P450_SCC_ (CYP11A1) are of significant interest. The detailed study of these plants showed for the first time that even the most specific component of the protein system of biosynthesis of animal steroid hormones is compatible with the hormonal system of plants and may be actively involved in its work, significantly improving the processes of growth, development and even plant immunity.

## Methods

### Plant material

Tomato seeds (*Solanum lycopersicum* L. cv. Rekordsmen) were kindly provided by the All-Russian Research Institute of Irrigated Vegetable, Melon and Ground Growing (Astrakhan oblast, Kamyziyak, Russia). The seeds were surface sterilized in 96% ethanol for 30 s and in 20% solution (*v*/*v*) of the commercial bleach ‘Ace’ with few drops of Tween-20 for 7 min, then rinsed with sterilized distilled water five times for 1 min each. After surface sterilization, the seeds were cultured on Murashige and Skoog (MS) basal medium [28] without growth regulators supplemented with 3% (*w*/*v*) sucrose and 0.7% (*w*/*v*) agar. The pH was adjusted to 5.8 before autoclaving. The cultures were maintained under 25 ± 1 °C, with fluorescence light (65 μmol m^−2^ s^−1^) during the long-day photoperiod (16 h light/8 h dark). The previously developed transgenic tobacco plants of the *N. tabacum cv. Petit Havana* SR1 line expressing full sized *CYP11A1* cDNA of cytochrome P450_SCC_ from the bovine adrenal cortex [29], the wild type plants and the plants transformed with an empty pGreen0229 vector [30], of new generations Т3, T4 and Т5 were cultivated at 25 °C with an illumination intensity of 1000–2000 lx and a photoperiod of 16 h.

### Phenotype analysis of transgenic tobacco plants

For comparative phenotypic analysis, we used Т4 and T5 generation plants. Seeds were sterilized at the surface and couched in a Murashige and Skoog medium [28] containing selective agent phosphinotricin. Phosphinotricin resistant plants were open field planted. All of the plants were tested for gene integration and expression by molecular genetic methods. During the course of the plants growth and development, the start time of flowering and different morphological parameters were checked. The study was performed on 15–25 independent plants of each line.

### Estimates of the speed of tobacco seeds sprouting and germination

Seeds germinated in Petri dishes on a filter paper. A Petri dish with slits was put on the grill with the tray. A strip of filter paper was passed through the slots at the bottom of the Petri dish providing the necessary hydration of the seeds. On the top Petri dishes was covered with caps to prevent seeds drying. Trays were incubated in a thermostat at 25 °C. In each variant there were 4 replications of 50 seeds. To determine the speed of germination number of germinated seeds was taken into account every day for 10 days. Seeds with the germ root of about 1 mm were counted as sprouted. Evaluation of germination was performed on the 10th day. Normally developed seedlings, the abnormal ones and not germinated seeds were taken into account. Statistical data processing was performed using software Microsoft Excel.

### Testing *CYP11A1* transgenic tobacco plants for resistance to fungal phytopathogen *Botrytis cinerea*

The phytopathogenic fungus of the species *Botrytis cinerea* causing gray mold was used as the test culture. In the provoking conditions, isolated leaves of medium age and of the same size were placed in Petri dishes on filter paper moistened with water. 10 μl suspension of spores of the pathogen containing 2000 spores/ml applied at 6 points on the sheet, after which the Petri dishes with leaves were tightly closed to create a high humidity and were placed and maintained in climate chamber at a temperature of 24 °C and 16-h photoperiod. The leaves of untransformed plants and plants containing only the empty vector plasmid (with no *CYP11A1* cDNA) were used as controls. The degree of damage of the leaves lamina, inversely proportional in relation to the relative resistance of the plant to *Botrytis cinerea* phytopathogen, were evaluated in points according to the number and size of necrotic spots on the 10th days after infection. Testing was also carried out by a different approach, i.e. by the method of counting the number of germinated spores in a drop of the juice from the leaves. 20 μl suspension of spores of *Botrytis cinerea* (10^5^ spores/ml) and 20 μl of untreated protein extracts from control (untransformed and transformed with an empty vector) and the *CYP11A1* transgenic plants were applied into microcuvettes that were subsequently incubated for 24 h at 24 °C. Counting of the number of germinated spores was carried out under the microscope.

### *Agrobacterium* strain and plasmid

**Fig. 1:**
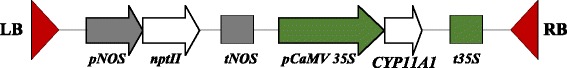
Schematic representation of the T-DNA region of the plasmid pBI121mod-P450_SCC_ used for tomato transformation. LB, left border; *pNOS*, nopaline synthase promoter; *nptII*, neomycin phosphotransferase II gene; *tNOS*, nopaline synthase terminator sequence; *pCaMV* 35S, constitutive promoter of 35S RNA of Cauliflower Mosaic Virus; *CYP11A1*, cDNA encoding cytochrome P450_SCC_ from the bovine adrenal cortex; *t35S*, 35S RNA terminator derived from Cauliflower Mosaic Virus; RB, right border

*Agrobacterium tumefaciens* strain AGL0 [31], carrying the binary vector plasmid pBI121mod-P450_SCC_ was used for tomato transformation. The T-DNA of plasmid pBI121mod-P450_SCC_ containing *CYP11A1* cDNA, encoding cytochrome P450_SCC_ from the bovine adrenal cortex, driven by the Cauliflower Mosaic Virus 35S promoter (*CaMV 35S*), as well as the neomycin phosphotransferase II (*nptII*) gene which confers resistance to kanamycin for selecting of putative transformants (Fig. 1). The plasmid pBI121mod-P450_SCC_ was introduced into *A*. *tumefaciens* strain AGL0 using the freeze-thaw method [32].

### *Agrobacterium-*mediated transformation of tomato

Cotyledons with petioles from 10 to 12-day-old tomato seedlings were used as explants for transformation. Prior to co-cultivation, the cotyledon explants were precultured for 3 days on agar-solidified MS medium containing 2 mg/l zeatin and 0.1 mg/l indole-3-acetic acid (IAA) with the abaxial surface in contact with the medium. Such hormonal treatments induced the highest shoots regeneration frequency from cotyledons of *tomato cv.* Rekordsmen [33].

*Agrobacterium tumefaciens* AGL0 harboring pBI121mod-P450_SCC_ binary vector was grown in a 250-ml capacity conical glass flask overnight at 28 °C in 50 ml of YEB liquid medium [34] containing 25 mg/l rifampicin and 50 mg/l kanamycin in a rotary shaker (150 rpm). *Agrobacterium tumefaciens* was resuspended at an OD_600_ = 0.6–0.7 with MS liquid medium. Then, pre-cultured explants were immersed in the above bacterial suspension for 20 min, blotted with sterile filter paper and transferred on Petri dishes containing agar-solidified MS medium, on the surface of which filter paper disks was placed. Co-cultivation was carried out at 16 °C in the dark for 72 h. After co-cultivation, the explants were washed with MS liquid medium supplemented with 300 mg/l timentin followed by rinsing five times to remove the bacterial overgrowth. Then the disinfected cotyledon explants were cultured on agar-solidified MS selection medium for callus induction containing 2 mg/l zeatin, 0.1 mg/l IAA, 25 mg/l kanamycin and 300 mg/l timentin. The explants were subcultured to fresh medium every 15 days. The explants survived in the selection medium generated calli from their cut ends. These calli were cultured in 300 cm^3^ glass cultures vessels containing medium for shoot regeneration (agar-solidified MS medium supplemented with 2 mg/l zeatin, 0.1 mg/l IAA, 25 mg/l kanamycin and 150 mg/l timentin). When regenerated kanamycin-resistant shoots were about 1.5-cm long, they were detached from the callus and transferred to root induction medium (half strength agar-solidified MS medium containing 0.2 mg/l indole-3-butyric acid and 100 mg/l kanamycin). Rooted independent transgenic lines of tomato were clonally multiplied in vitro and adapted to soil. Transgenic and non-transgenic tomato plants were grown in plastic pots filled with sterilized soil, at standard agricultural conditions (22–25 °C day time temperature and 18–19 °C night time temperature, humidity 60–70%, illumination 2500 lx). Plants were propagated by grafting of lateral shoots formed on the adult plants.

### DNA/RNA extraction, PCR analyses and other molecular genetic techniques

Total genomic DNA was isolated from leaves of putative transformants and wild-type (WT) tomato plants according to Edwards et al. [35] with additional extraction with saturated phenol. To determine the quality of the isolated DNA for PCR analysis, the sequence of the *Tom52* actin gene of tomato (NCBI, U60482) was amplified. The amplification was also carried out on the sequence of the selective (*nptII*) and target (*CYP11A1*) genes, as well as the virulence genes of the *Agrobacterium tumefaciens* (*virB*). Primer sequences, PCR conditions and the expected size of amplified products are presented in Additional File 2. The PCRs were carried out in a 25-μl volume containing 2.5 μl of 10X PCR buffer, 0.5 μl of 10 mM dNTPs, 1 μl of forward and reverse primers at 10 qM, 1 μl of 5 Uμl^−1^ Taq polymerase, 17 μl of deionized water and 2 μl (~60 ng) of a DNA template. PCR products were carried out in a MJ Mini Personal ThermalCycler (Bio-Rad). The products were separated in 1.0% agarose gel in an electrophoresis system (Amersham Electrophoresis Power Supply—EPS 301 + Hoefer HE 33 Mini Horizontal Submarine Unit).

The expression of *CYP11A1* cDNA in transgenic tomato plants was assessed using RT-PCR technique (PCR coupled with reverse transcription). Isolation of total cell RNA from leaves of 5-week-old plants grown in a phytotron was carried out according to [36] or by using kit Total RNA Purification Kit (Jena Bioscience). cDNA was synthesised on the RNA template using the Maxima First Strand cDNA Synthesis Kit for RT-qPCR (Thermo Scientific). PCR with synthesized cDNA was performed with primers complementary to the sequences of the target gene *CYP11A1*, the size of the expected PCR fragment was 520 bp [37]. A fragment (709 bp) of the gene encoding large ribosomal subunit of tomato (25S rRNA) [38] was used as a “housekeeping” gene. cDNA isolated from the original Rekordsmen variety was used as negative control. Standard procedures of molecular cloning were used for recombinant plasmid constructions [34].

### Transmission electron microscopy (TEM)

The seeds of wild-type tobacco plants (*Nicotiana tabacum* cv. Petit Havana SR1) and two transgenic homozygous tobacco lines (TR-2 and TR-7) of T4 generation expressing *CYP11A1* cDNA, encoding cytochrome P450_SCC_ from the bovine adrenal cortex, were aseptically germinated on the MS basal medium [28] supplemented with 3% (*w*/*v*) sucrose and 0.7% (w/v) agar. The middle sections of cotyledons from the seedlings were fixed for 24 h in 2.5% glutaraldehyde (Merck, Germany) dissolved in 0.1 M Sorensen’s phosphate buffer (pH 7.2) with 1.5% sucrose. Then the samples were washed, postfixed in 1% ОsО4 (Sigma-Aldrich, USA), dehydrated in ethanol of increasing concentrations (30, 50, 70, 96, and 100%) and in propylene oxide (Fluka, Germany). The samples were embedded in mixture of Epon-812 and Araldite (Merck, Germany) according to the standard procedure. For TEM the embedded samples were sectioned with diamond knife using ultramicrotome LKB-V (LKB, Sweden), placed on formvar coated grids and stained with uranyl acetate and lead citrate [39]. The ultrathin sections were examined and photographed with electron microscope Н-500 (Hitachi, Japan). Ultrastructure of mitochondria in mesophyll cells was studied. The average cross-sectional area of mitochondria was determined using the Cell-A software (Olympus, Japan). At least 200 mitochondria were scored for each treatment.

### The study of the transgenic tomato plants resistance to drought

It consisted of two stages: study of the reaction of samples on the long absence of irrigation and investigation of the recovery of plants after the resumption of irrigation. The experiment was carried out in the conditions of the glass box in the absence of irrigation, accompanied by high day (35–40 °C) and night (14–16 °C) temperatures. Plants in the development stage of 3–4 true leaves were grown in the absence of irrigation for 3 weeks, biometric indicators were recorded at 7th, 14th and 21th days. Biometric parameters (plant height; mean increment; the number of plants, wilted on 50 and 100%) of transgenic lines No. 4 and No. 7 were compared with each other and with the parent variety Recordsmen grown in the same conditions. Sample size: 15 plant of the variety Recordsmen, 60 plants of T2 generation of the transgenic lines No. 4 and No. 7.

### Isolation of steroid fractions from tobacco and tomato leaves

Tobacco and tomato leaves were frozen in liquid nitrogen and lyophilized. Total lipids were extracted from 0.1 g of dry plant material by a CHCl_3_–MeOH–H_2_O mixture (5: 10: 4) 3 times for 4 ml. Extracts were joined, dried using a rotary evaporator at 85 °C, and separated by TLC using plates with silica gel 60 F 254 by Merck (200 × 200 mm, 0.5 mm thick) in a CHCl_3_–EtOAc system (4:1). One part of the plate was colored by a reagent with anisaldehyde (0.5 ml of anisaldehyde in 10 ml 98% H_2_SO_4_, 85 ml MeOH, and 5 ml 96% H_2_SO_4_). Zones that coincided by chromatographic mobility with cholesterol, pregnenolone, and progesterone were scratched out of the uncolored part of the plates, extracted by CHCl_3_, and dried using a rotary evaporator. The dry residue was dissolved in MeOH.

### Determination of the progesterone content in transgenic plants

It was carried out using a combination of the TLC method and immunoenzymatic assay. Progesterone enzyme immunoassay was applied to leaf extracts from transgenic tobacco and tomato plants, wild-type plants, and to fractions, the chromatographic mobility of which under purification by TLC coincides with the chromatographic mobility of progesterone, according to the service instructions (the recommendations of the kit manufacturer) for the PROGESTERONE EIA kit. The kit was developed at the Institute of Bioorganic Chemistry, the National Academy of Sciences of Belarus, and is intended for quantitative progesterone determination in human blood serum by enzyme linked immunosorbent assay. The kit was kindly provided by A.G. Pryadko.

## Results

### Phenotype of transgenic tobacco plants expressing mammalian *CYP11A1* cDNA

#### The accelerated growth and development of the T4 generation of transgenic tobacco plants expressing mammalian *CYP11A1* cDNA

**Fig. 2:**
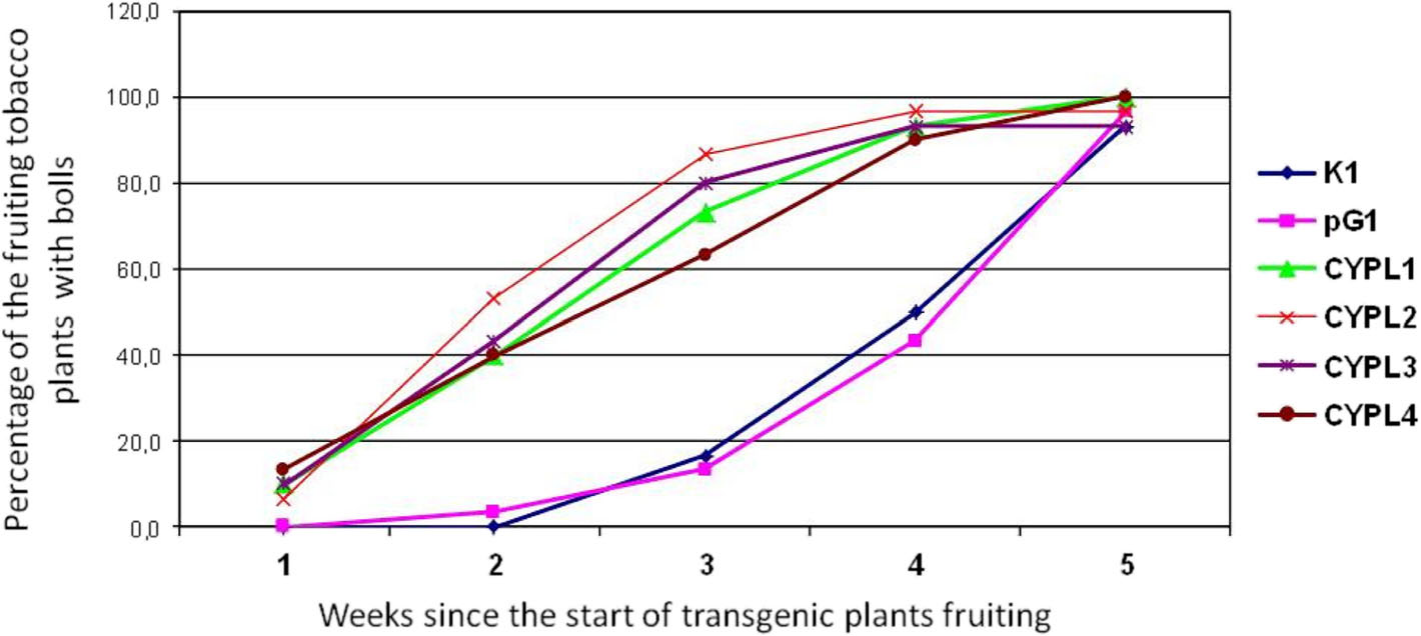
Dynamics of bolls’ formation of transgenic tobacco plants of T4 generation. CYPL1–CYPL4 – independent lines of transgenic tobacco plants expressing *CYP11A1* cDNA; K1 – control plants of wild type; pG1 – plants transformed with an empty vector (not containing *CYP11A1* cDNA)

**Fig. 3:**
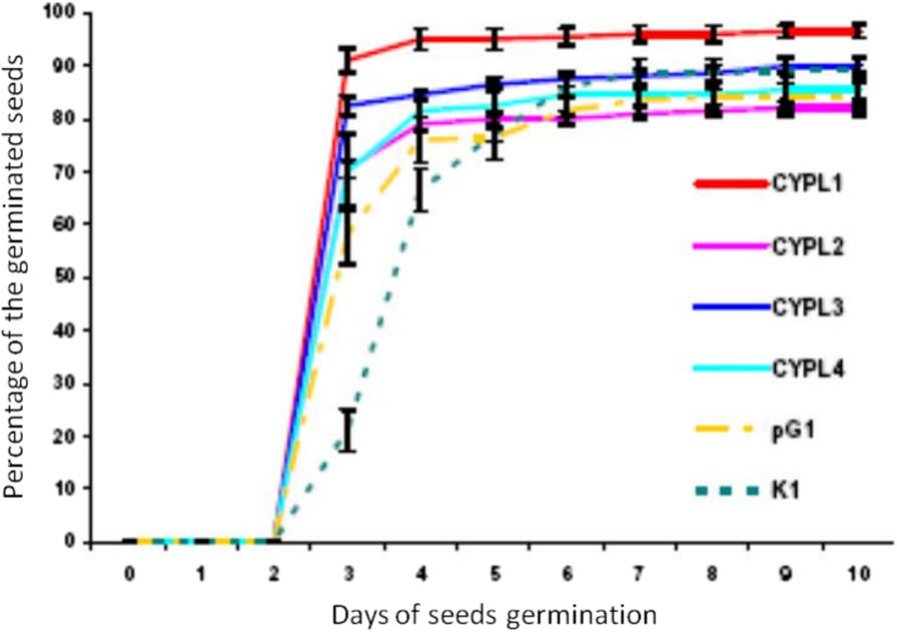
Dynamics of seeds germination of transgenic tobacco plants of the T4 generation. CYPL1–CYPL4 – independent lines of transgenic tobacco plants expressing *CYP11A1* cDNA; K1 – control plants of wild type; pG1 – plants transformed with an empty vector (not containing *CYP11A1* cDNA)

We have continued to study the *CYP11A1* transgenic *Nicotiana tabacum* plants described by us earlier [29]. As with previous generations of transgenic lines CYPL1-CYPL4, plants of the T4 and T5 generations by their habitus was significantly superior to control plants already in the early stages of development (Additional File 3). Moreover, as noted by us earlier for previous generations [37], transgenic tobacco lines CYPLs begun flowering on average 2 weeks earlier than control plants and the line of the transformants pG1. Formation of bolls in all lines of transgenic plants of T4 generation did also started at least a week earlier compared to the control plants and plants with empty vector (Fig. 2). Seeds of all lines of transgenic plants of the T4 generation (CYPL1-CYPL4) and control plants (K1) and plants with empty vector (pG1) was tested for germination according to methodology described in Methods. Such indicators of physiological seed quality as vigour of seeds sprouting, which characterizes the ability of seeds to germinate quickly and about together (at the same time) have been evaluated. The energy of germination was determined on the third day. It was noted that on the third day in all lines of the *CYP11A1* transgenic plants had sprouted from 70 to 91% of seeds (lines CYPL1–91%, CYPL2–82.5%, CYPL3–70.5%, CYPL4–70%), and in control variants (line K1 and the line pG1 with an empty vector) – only 21% and 58%, respectively (Fig. 3).

Table 1 summarizes the major morphological characteristics of the T4 generation of the studied lines of independent transgenic and control plants. The data presented show that all lines of the *CYP11A1* transgenic tobacco plants significantly differ from the control wild type and plants transformed with an empty vector by such important physiological indicators as plant height, diameter of stem, size of leaves, number of bolls and weight of thousand seeds (Table 1).

**Table 1:** Morphological features of the transgenic tobacco plants of T4 generation

Plant line	Plant height, cm*	Stem diameter, mm*	Leave square, cm^2^*	Number of bolls (seed pods), pieces*	Weight of 1000 seeds, mg*
CYPL1	139 ± 12	1.74 ± 0.42	480 ± 65	63 ± 14	69.8 ± 1.9
CYPL2	132 ± 14	1.82 ± 0.43	425 ± 87	51 ± 21	80.2 ± 0.9
CYPL3	130 ± 11	1.87 ± 0.60	338 ± 58	80 ± 14	65.2 ± 1.5
CYPL4	124 ± 12	1.96 ± 0.50	610 ± 102	58 ± 20	80.5 ± 1.3
pG1	89 ± 24	1.49 ± 0.28	210 ± 31	39 ± 13	50.3 ± 0.9
K1	87 ± 15	1.24 ± 0.28	241 ± 38	39 ± 13	50.3 ± 1.7

CYPL1–CYPL4 – independent lines of transgenic tobacco plants expressing *CYP11A1* cDNA; pG1 – plants transformed with an empty vector (not containing *CYP11A1* cDNA) and K1 – control plants. The data represent average values (± standard deviation) for the *n* = 30 samples

*All these differencies between transgenic (CYPL1-CYPL4) and control (K1 and pG1) lines are statistically valid (*P* < 0,05)

#### Transgenic tobacco plants expressing mammalian *CYP11A1* cDNA are resistant to infection by fungal pathogens *Botrytis cinerea*

**Fig. 4:**
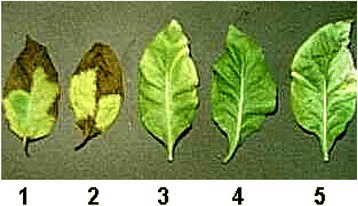
Results of the bioassay on resistance of transgenic tobacco plants expressing cDNA of the *CYP11A1* gene to a fungal phytopathogen *Botrytis cinerea*. 1 – control nontransgenic (WT) plant (infection spread - 7 points); 2 – control transgenic plant transformed with empty vector (infection spread - 7 points); 3–5 – independent lines of transgenic plants, expressing cDNA of *CYP11A1* gene (3 – infection spread - 1 point; 4 – infection spread - 0 point; 5 – infection spread - 2 points)

Testing of the transgenic tobacco plants expressing *CYP11A1* cDNA of animal origin on resistance to nonspecific *Solanaceae* pathogen, fungus *Botrytis cinerea*, was carried out both on isolated leaves (Fig. 4), and by the method of counting the number of germinated spores in a drop of the juice from the leaves. The results obtained are given in the Additional File 4 and show that while the arithmetic means of the number of germinated spores for the control transgenic (with empty vector) and a non-transgenic tobacco lines are varying from 80 to 96%, for the transgenic tobacco lines expressing the cDNA of mammalian *CYP11A1* gene the number of germinated spores are much lower (ranging from 7 to 21%). These results indicate the suppression of growth of pathogenic fungi by protein extracts isolated from transgenic plants that contain the gene under study. This is also evidenced from the results of the bioassay for resistance to *Botrytis cynerea*, based on the elucidation of the size and number of necrotic spots on plant leaves 10 days after infection, represented in Fig. 4. In stable transgenic lines of tobacco expressing cDNA of *CYP11A1* gene, the average areas of affected sites on the leaves on 10th days after infection were approximately 12 times smaller than that in control plants. In some cases the areas of necrosis on the *CYP11A1* transgenic leaves were completely absent.

#### Transmission electron microscopy (TEM) of the cotyledon leaves of control and *CYP11A1* transgenic tobacco plants and the ultrastructure of their mitochondria

**Fig. 5:**
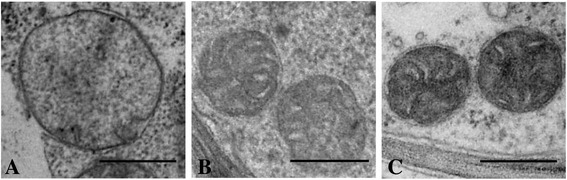
Mitochondrial ultrastructure of cotyledonary mesophyll cells from the seedlings of wild type tobacco plants (**a**) and transgenic tobacco lines TR-2 (**b**) and TR-7 (**c**) expressing mammalian *CYP11A1* cDNA. Bar – 0.25 μm

**Fig. 6:**
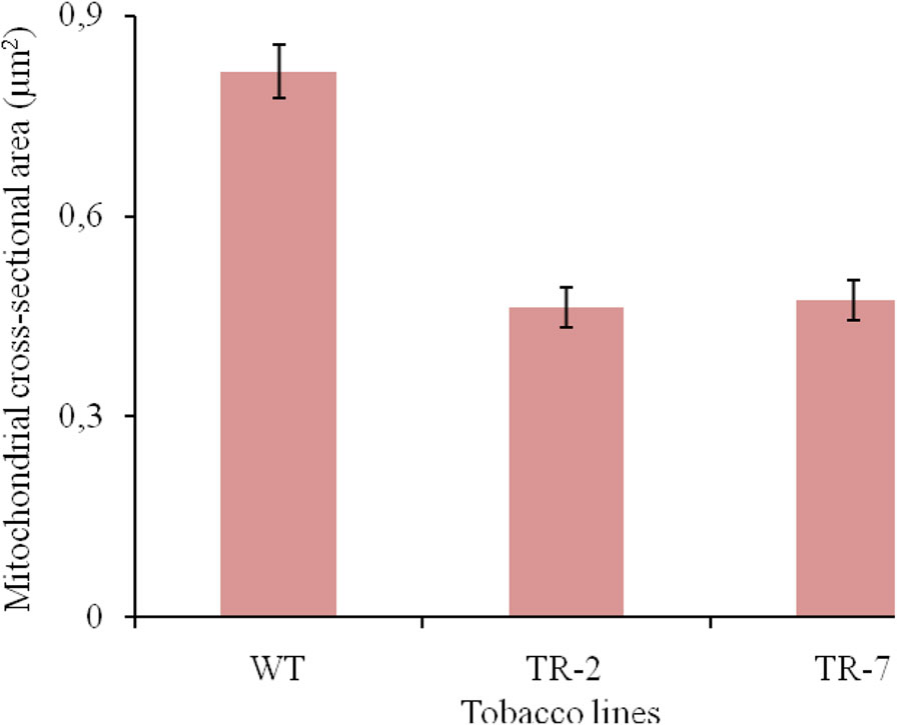
The average cross-sectional area of mitochondria in cotyledonary mesophyll cells of wild type (WT) and *CYP11A1* transgenic (TR-2 and TR-7) tobacco plants

TEM results revealed significant differences in the size and structure of mitochondria from the mesophyll cells between wild-type and transgenic tobacco plants. In wild-type plants, mitochondria were round or oval. Mitochondria with double membrane, electron light matrix and few peripherally localized and disordered cristae are clearly visible (Fig. 5a). The average cross-sectional area of mitochondria from the mesophyll cells of wild-type tobacco plants was 0,817 μm^2^ (Fig. 6). Unlike wild-type plants, the mitochondrial cross-sectional areas of transgenic tobacco line TR-2 and TR-7 were 1.8 and 1.7 times less, respectively. There was no significant difference in the mitochondrial cross-sectional areas between transgenic lines expressing *CYP11A1* cDNA. The evenly distributed, round or elongated mitochondria are predominant. They are characterized by electron-dense matrix, as well as the large number of well-formed and enlarged cristae, some of which are parallel to each other (Fig. 5b, c).

### Construction of transgenic tomato plants expressing mammalian *CYP11A1* cDNA

#### Tomato transformation

**Fig. 7:**
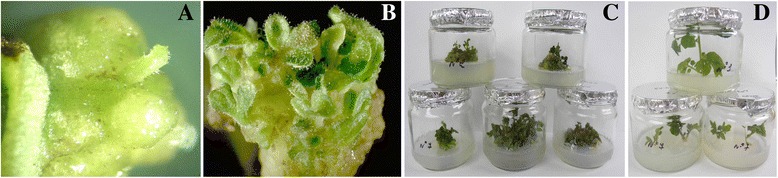
In vitro callus induction and shoot regeneration of Rekordsmen tomato plants from cotyledons inoculated with *Agrobacterium tumefaciens* harboring the pBI121mod-P450_SCC_ binary vector. (**a**) Callus formation from cotyledonary explants on selection medium supplemented with 25 mg/l kanamycin after 15–25 days of culture, (**b**) Shoot regeneration from transformed callus on selection medium after 6 wk. of culture, (**c**) Mass regeneration of tomato shoots from transformed callus, (**d**) Well-rooted plantlets on root induction medium supplemented with 100 mg/l kanamycin

Two independent experiments were carried out to produce transgenic tomato plants containing the *CYP11A1* cDNA. A total of 460 pre-cultured explants were used for *Agrobacterium*-mediated transformation. Figure 7 illustrates the different stages of callus formation and shoot regeneration from cotyledon explants inoculated with *Agrobacterium tumefaciens* strain AGL0 harboring the pBI121mod-P450_SCC_ plasmid. Compact and light green callus began to form on the cut-edge of the explant 2 to 3 weeks after transformation (Fig. 7a). Explants which did not produce callus were discarded. The frequency of callus formation from transformed explants on selection medium supplemented with 25 mg/l kanamycin was 5.2%. Actively growing callus is excised from the explant and subcultured separately on fresh culture medium as above. Shoots regeneration from callus was observed after 6 weeks of culture (Fig. 7b). Through more than 2 month of selection, 8 kanamycin-resistant calli with multiple shoot regeneration were obtained (Fig. 7c). The elongated shoots when they were 1.5 cm long were cut off and transferred onto root induction medium supplemented with 100 mg/l kanamycin. All independent putative plantlets produced roots within 1–2 weeks (Fig. 7d). Rooted tomato plantlets were clonally multiplied in vitro and adapted to soil.

#### PCR analysis of putative transgenic tomato plants

**Fig. 8:**
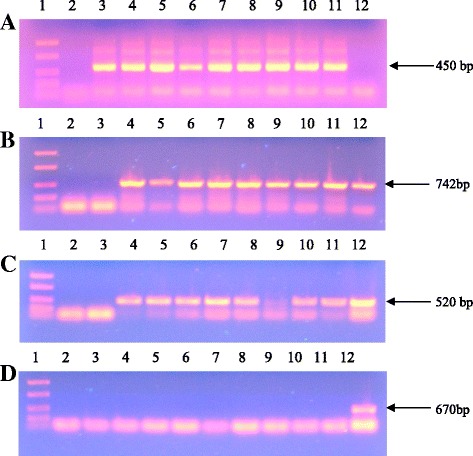
PCR amplification of the *Tom52* gene (**a**), *nptII* gene (**b**), *CYP11A1* gene (**c**) and *virB* gene (**d**) from total genomic DNA of putative transformants and wild type (WT) tomato plants. Line 1: FastRuler™ Low Range DNA ladder (Fermentas, Lithuania), lanes 2–3: water and non-transformed tomato as negative controls, lanes 4–11: independent transformed tomato lines, line 12: positive control (pBI121mod-P450_scc_ plasmid)

Total genomic DNA was isolated from the young leaves of all kanamycin resistant tomato plants as well as wild-type (WT) tomato. To assess the suitability of the isolated plant DNA for PCR analysis, the sequence of the *Tom52* was amplified (Fig. 8a). An 742 bp fragment was observed from all tested putative tomato transformants with specific primers for the *nptII* gene, whereas no corresponding band was detected in the untransfomed plant (Fig. 8b). Out of 8 transgenic plants, 7 were found with integration of the target gene into the *Solanum lycopersicum* L. genome (Fig. 8c). To exclude the possibility of *Agrobacterium* contamination in transformed plants, total DNAs were analysed with PCR using specific primers for *virB* gene. Contamination by the *Agrobacterium virB* gene was not detected in the analyzed samples (Fig. 8d). Based on the results of PCR analysis, we calculated transformation efficiency, which was 1.5%. Transformation efficiency was assessed as the percentage of independent transgenic tomato lines, containing the selective and target genes, per total number of transformed explants.

**Fig. 9:**
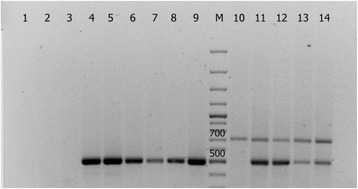
RT-PCR of cDNA of tomato plants with primers specific for the *CYP11A1* (520 bp) and *25S RNA* (709 bp) genes. 1–3, 10 – control plants; 4–6, 11, 12 – individual plants of the line No. 4; 7–9, 13, 14 – individual plants of the line No. 7

The presence of *CYP11A1* cDNA expression in transgenic tomato plants was assessed using RT-PCR (PCR with reverse transcription) (see Methods). The results which are shown on Fig. 9 clearly indicate that all represented individual plants of the lines No. 4 and No. 7 efficiently express mammalian *CYP11A1* cDNA, and expression of this heterologous coding sequence in line No. 4 is somewhat higher than in line No. 7 (as judged by comparison of the multiplex PCR bands’ intensities in lanes 11–12 & 13–14). This could probably indicate double insertion of *CYP11A1* cDNA in the transgenic tomato line No. 4.

### Phenotype of transgenic tomato plants expressing mammalian *CYP11A1* cDNA

#### Common and specific features of phenotype of the T3 generation of transgenic tomato plants (lines No. 4 and No. 7) expressing mammalian *CYP11A1* cDNA

Both transgenic tomato lines (No. 4 and No. 7), containing in their genomes mammalian *CYP11A1* cDNA that is stably transmitted in seed breeding, have revealed some general patterns of onset and phenotypic differences in growth, development and formation of fruits in comparison with the original tomato variety Recordsmen. Overall for transgenic lines was an impaired development of the generative system (impaired development of flower brushes, low fruits set). Part of the buds were falling after flowering (Additional File 5), some samples formed atypically large sepals and in some cases the absence of stamens and pistils was observed. Seeds formed in the fruits were different in line No. 4 and line No. 7. A significant part of the fruits of the line No. 7 was seedless or only with the seed germ, while the fruit of the line No. 4 showed normal seed formation (Additional File 6). The reduction in fertility of plants of the transgenic tomato lines is probably due to the impaired development of anthers, which in turn may be a result of the reduction of amount of (24R)-brassinosteroids, as we previously identified in the case of *CYP11A1* transgenic tobacco plants [11, 12, 37]. And the other common feature of the two tomato transgenic lines was the increased resistance to such abiotic stresses as drought and dehydration (see next section).

Interestingly, the two different valuable phenotypes, that we noted in *CYP11A1* transgenic tobacco plants, were clearly separated in the two transgenic tomato lines we have obtained: one line (No. 4) have an accelerated rate of vegetative development (Additional File 7), while the other (No. 7) have enhanced immunity to abiotic and biotic stresses. Plants of the line No. 7 are characterized by high resistance to stress loads what can be seen already from intense green color of their leaves and stem branches and large leaf blades with corrugated surface (Additional File 8) and especially by their intensive growth at the end of the vegetative season (see Table 2).

**Table 2:** The growth characteristic of the transgenic tomato plants on different ontogenesis stages

	Plant height			Growth increment		
	June, cm	July, cm	September, cm	June–July, cm	July–September, cm	Total (from June until September), cm
Recordsmen (mean)	79.9	138.1	139.1	58.2	1.0	59.2
Line No. 4 (average)	60.0	112.8	126.5	52.8	13.7	66.5
Line No. 7 (average)	57.0	114.1	158.4	57.1	44.3	101.4

#### Both transgenic tomato plants lines (No. 7 & No. 4), expressing mammalian *CYP11A1* cDNA, are more resistant to drought and prolonged dehydration in comparison with wild type variety Recordsmen

Growing of control and transgenic tomato plants under drought and dehydration conditions showed that tomato plants during 3 weeks have demonstrated the growth in height on 2–3 cm, despite the lack of watering. Maximum plant growth was observed for the line No. 7, with individual plants reaching up to 7–12 cm. The height of plants of the control variety Recordsmen and some plants of the line No. 4 for 3 weeks remained at the same level. All the studied plants had different degree of wilting of the leaves and the shoot apex. By the end of the 3rd week of the experiment, the maximum number of wilted plants (33%) was observed in the cultivar Recordsmen and among the plants of the line No. 4 (10%). Plants of the line No. 7 were the most resistant to the lack of irrigation, and were characterized by a slight wilting of 3 from 15 plants investigated, while no plant died (Table 3).

**Table 3:** Biometric indicators of the reaction on prolonged drought of the control (cultivar Recordsmen) and two independent homozygous transgenic lines No. 7 (7–15, 7–21, 7–27) and No. 4 (4–18, 4–34) of tomato *Solanum lycopersicum* L. plants of generation T2

Plant lines	Medial plant height, cm		Mean increment, cm	Number of plants, wilted on 50%	Number of plants, wilted on 100%
Control	7 days	17.4 ± 0.8			
	21 days	18.3 ± 0.8	0.9	5	7
7–15	7 days	12.9 ± 0.8			
	21 days	15.9 ± 0.7	3.0	8	0
7–21	7 days	15.7 ± 0.7			
	21 days	17.8 ± 0.8	2.1	10	2
7–27	7 days	11.5 ± 0.7			
	21 days	16.9 ± 1.0	5.4	3	0
4–18	7 days	12.0 ± 0.8			
	21 days	14.2 ± 0.7	2.2	5	2
4–34	7 days	14.1 ± 0.5			
	21 days	15.3 ± 0.5	1.2	3	6

Thus, both transgenic lines showed a lower degree of exposure to drought and showed increased resistance to no irrigation for 3 weeks, compared to the control sort Recordsmen. Most of the plants of transgenic lines exceeded the Recordsmen’ plants for the growth of the stem during the drought period: in plants of the line No. 7 stem height increased by more than 3 cm, in plants from the line No. 4 – by 1.8 cm, while the average stem growth in plants of the control variety Recordsmen was 0.9 cm (Table 3). Similarly, more active regeneration and enhancement of growth was observed in the lines No. 7 and No. 4 after the resumption of irrigation.

#### ELISA determination of progesterone in transgenic tobacco and tomato plants

**Fig. 10:**
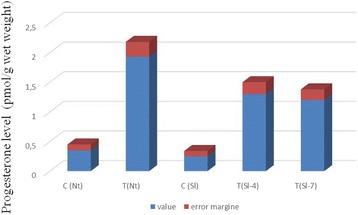
Immunoenzymatic analysis of steroid fractions of tobacco and tomato leaves C(Nt) – control (WT) tobacco plants; T(Nt) – transgenic tobacco plants; C(Sl) – control (WT) tomato plants; T(Sl-4) – transgenic tomato plants, line No. 4; T(Sl-7) – transgenic tomato plants, line No. 7.

We determined the relative content of progesterone in the extracts from the control and transgenic tobacco and tomato plants using ELISA. To conduct this analysis, we chose one line of transgenic tobacco (TR-7) and two independent lines of transgenic tomato plants (No. 4 and No. 7), all stably inheriting and expressing the *CYP11A1* transgene, and all showing clear differences in their phenotype from the control plants for most of the studied parameters. Since plants may contain steroid compounds that give cross-reactions with antibodies to progesterone, we determined its content not only in total extracts from leaves of plants, but also in the fractions with chromatographic mobility identical to progesterone after separation of the total extracts by TLC. It turned out that in both cases, the content of progesterone in leaves of transgenic plants is 3–5 times higher than its content in the leaves of plants of wild type (Fig. 10).

## Discussion

To more thoroughly compare steroidogenic systems of plants and animals, we have constructed and analyzed transgenic tobacco and tomato plants expressing *CYP11A1* cDNA encoding bovine mitochondrial cytochrome P450_SCC_. Through five consecutive generations transgenic tobacco plants expressing heterologous sequence of the mammalian *CYP11A1* cDNA have a distinct, clearly distinguishable phenotype characterized by the reduced period of vegetative development (early flowering and maturation of bolls), enlarged biomass and increased productivity (quantity and quality of seeds), and also greater resistance to biotic stresses: positive effect of *CYP11A1* on resistance of transgenic tobacco plants to the infection by fungal phytopathogen *Botrytis cinerea* was for the first time detected. Subtle changes in mitochondria of the transgenic *Nicotiana tabacum* plants expressing mammalian *CYP11A1* cDNA were demonstrated by transmissive electron microscopy indicating the successful incorporation of the mammalian cytochrome P450_SCC_ into plant mitochondria, similarly to the situation previously shown by in vitro experiments [40]. This is the first direct evidence that cytochrome P450-dependent monooxygenase can be incorporated into the mitochondria of living plant cell with the ability to successfully operate in it.

Together with previously published data of other authors on profound relatedness of electron transfer chains of P450-dependent monooxygenases in mammalian and plant mitochondria (the amino acid residues responsible for protein-protein interactions are fully conserved in plant MFDXs and MFDR proteins [21–24]), the results obtained support our previous findings about functional compatability of Plantae and Animalia steroidogenic systems [37] and indicate the existence in plants of another steroid regulatory system in addition to brassinosteroids. Moreover, our previous results about the decreased level of 24R–brassinosteroids in seeds of transgenic tobacco plants expressing *CYP11A1* cDNA [37] indicate that brassinosteroids’ and progesterone’ hormonal systems of plants in some cases can act as antagonists having opposite effects on the activity of plant cells and physiological processes in plants.

Although the only currently known process in plants, which involves mitochondrial ferredoxins (AtMFDX1 and AtMFDX2) and mitochondrial ferredoxin reductase (AtMFDR) is the final stage in the synthesis of vitamin B8 (biotin) [22], these data of *C. Alban’s* group and the analysis of the proteome of plant mitochondria [41] clearly indicate that at least MFDX1, MFDX2 and MFDR of *Arabidopsis* and potato are localized and function in the plant mitochondria, and thus are able to maintain the mitochondrial P450 reactions. Thus, we for the first time proved that individual components of biosynthesis systems of animal and plant steroid hormones are compatible with each other in vivo and can work together, significantly improving the processes of growth, development, and even plant immunity. These results are in good agreement with earlier and more recent studies of other authors investigating the influence of exogenous progesterone on the physiology and reproduction of plants. In particular, these works showed that progesterone even at very low concentrations stimulates maturation of pollen and the growth of pollen tube in tobacco [42], induces flowering and generative development of wheat and *Arabidopsis*, and speeds up in the light and in the dark periods the growth of *Arabidopsis thaliana* seeds [10], enhances the growth of *lh*-lines of peas with a mutation in the gibberellin(s) [9], stimulates the activity of antioxidant enzyme catalase from chickpeas [43], inhibits the growth of many pathogenic and saprophytic bacteria and fungi, such as *Rhizopus nigricans* [9, 10].

It should be noted that a number of animal steroid hormones (pregnenolone sulfate, progesterone, 17-hydroxyprogesterone, 16-dehydroprogesterone, androstenedione) and receptors which mediate mitochondrial cholesterol uptake in animal cells have also been recently discovered in many divergent plant species [9, 26, 27] (Additional file 1). As can be seen from the foregoing scheme of reactions, the biosynthesis of all these steroid hormonal compounds could be easily derived from the four main plant sterols (β-sitosterol, stigmasterol, campesterol and cholesterol) via pregnenolone and progesterone as the key steroid regulators. From earlier data it is known that progesterone was found in many different plants (*Arabidopsis*, tomato, potato, rice, pea, apple seeds, *Holarrhena*, *Strophanthus*, *Nerium*, *Digitalis*, *Punica*, *Erysimum*, *Haplopappus*, etc.) [9, 44, 45] from various families (*Apocynaceae*, *Scrophullareaceae*, *Lythraceae*, *Brassicaceae*, *Astraceae*, etc), which testifies to its probable presence in all plants. With the regard to all the data and the fact that a membrane progesterone binding protein (MSBP1), which is a homologue of an animal membrane progesterone binding protein, was found in *Arabidopsis thaliana* [46], the progesterone could be regarded as a new real hormone of the plant cell forming a core of novel, but very ancient by its evolutionary origin, regulatory steroid system in plants. By means of binding to MSBP1 as a cell membrane receptor, progesterone not only takes part in regulation of plant growth and development, but probably is also responsible for the quick response to various external stimuli, such as different abiotic and biotic stresses.

At the same time, even our purely bioinformatic analysis shows that hormonal ‘progesterone’ system of plants and animals have important differences. Although both plants and animals have the membrane progesterone receptors represented by the family of membrane steroid-binding protein (MSBP) [46] or MAPR (membrane-associated progesterone receptors), nuclear receptors of progesterone with the much greater binding capacities and specificity are present only in animals. Although may be a homologue of the estrogen receptor (estrogen receptor, ER), the evolutionarily very first of the mammalian nuclear steroid hormone receptors, is present at least in some plants, such as *Solanum glaucophyllum* [47].

## Conclusion

The compatibility in vivo of even the most specific components of biosynthesis systems of steroid hormones in Plantae and Animalia was demonstrated for the first time. Cytochrome CYP11A1 (P450_SCC_), a key enzyme of animal steroidogenesis, can function in transgenic (expressing mammalian *CYP11A1* cDNA) *CYP11A1* tobacco and tomato plants accelerating the processes of growth and development and enhancing the plants resistance to biotic and abiotic stresses. The hypothesis is proposed and substantiated that the formation of the above-noted special phenotype of transgenic plants expressing mammalian *CYP11A1* cDNA is due to the increased biosynthesis of progesterone, which can be considered as a very ancient bioregulator of plant cells and the first hormone common to plants and animals. The results indicate a definite similarity (truly fundamental kinship) of the steroid compounds biosynthesis and steroid regulatory systems of plants and animals and can be used in new biotechnologies for agriculture and pharmacology.

## Additional files

Additional file 1: Scheme of biosynthesis of the detected mammalian steroid hormones in plants. (DOC 74 kb)

Additional file 2: Description of primers, conditions of amplification and product length for PCR analyses. (DOC 34 kb)

Additional file 3: Control (1, hight – 15 cm) and transgenic (2, hight - 72 cm) tobacco plant after one month of growth in the field conditions. (DOC 157 kb)

Additional file 4: Assessment of fungicidal properties of transgenic tobacco plants. (DOC 28 kb)

Additional file 5: Compound racemes with large sepals and undeveloped pistils and stamens of the transgenic line No. 7. (DOC 237 kb)

Additional file 6: Fruit size and seed formation of the transgenic lines No. 4 and No. 7. (DOC 633 kb)

Additional file 7: Growth stages of the *CYP11A1* transgenic tomato plants in the second decade of September. In the foreground – line No. 4 followed by the control variety Recordsmen (far behind), on the left side – line No. 7. Note the accelerated course of ontogenesis stages in the line No. 4 (as a result – precocity of some fruits and yellow leaves). Line No. 7 is characterized by greening and the intensive growth at the end of the vegetative season. (DOC 107 kb)

Additional file 8: Specific features of the *CYP11A1* transgenic line No. 7: an intense green color of leaves and stem branches, large leaf blades with the corrugated surface, low amount of seeds in fruits, increased resistance to diseases. (DOC 72 kb)

## Abbreviations

5αR: 5α-reductase
Ad: Adrenodoxin
AdR: Adrenodoxin reductase
CST: Cytoplasmic sulfate transferase
CYP: Cytochrome P450
DET2: De-etiolated 2
DHP: Dihydroprogesterone
HSD: Hydroxysteroid dehydrogenase
MFDR: Mitochondrial-type ferredoxin reductase of plants
MFDX: Mitochondrial-type ferredoxins of plants
P450_SCC_: P450 side chain cleaving
pCaMV 35S: the Cauliflower Mosaic Virus 35S promoter
TEM: Transmission electron microscopy
WT: Wild type
