# Supplementary material for: A key enzyme of animal steroidogenesis can function in plants enhancing their immunity and accelerating the processes of growth and development
Source: BMC Plant Biol. 2017 Nov 14;17(Suppl 1):189. doi: 10.1186/s12870-017-1123-2 (PMC5688476; doi:10.1186/s12870-017-1123-2)
Supplement: Supplementary file 1 — Scheme of biosynthesis of the detected mammalian steroid hormones in plants. (DOC 74 kb) [file 12870_2017_1123_MOESM1_ESM.doc]

Additional file 1. Scheme of biosynthesis of the detected mammalian steroid hormones in plants.
