# Supplementary material for: A key enzyme of animal steroidogenesis can function in plants enhancing their immunity and accelerating the processes of growth and development
Source: BMC Plant Biol. 2017 Nov 14;17(Suppl 1):189. doi: 10.1186/s12870-017-1123-2 (PMC5688476; doi:10.1186/s12870-017-1123-2)
Supplement: Supplementary file 2 — Description of primers, conditions of amplification and product length for PCR analyses. (DOC 34 kb) [file 12870_2017_1123_MOESM2_ESM.doc]

**Additional File 2. Description of primers, conditions of amplification and product length for PCR analyses.**

| Gene | Primer Sequence (5' → 3') | PCR conditions | Product length (bp) |
| --- | --- | --- | --- |
| *Tom 52* | forward: GCCAGGTATTGTGCTGGACT  reverse: ATCAGCAATACCAGGGAACA | Initial denaturation for 5 min at 94°C;  35 cycles of denaturation at 95°C for 30 sec, annealing at 54°C for 30 sec, and elongation at 72°C for 30 sec; final elongation for 5 min at 72°C | 450 |
| *CYP11A1* | forward: GAGGAACCGCAGGAGTCGA  reverse: GGAGAAACCCACGTCATGCG | Initial denaturation for 5 min at 94°C;  35 cycles of denaturation at 95°C for 30 sec, annealing at 60°C for 45 sec, and elongation at 72°C for 45 sec; final elongation for 5 min at 72°C | 520 |
| *nptII* | forward: CGCGGGTTTCTGGAGTTTAATGAGCTAAG  reverse: GCATGCGCGCCTTGAGCCTGG | Initial denaturation for 5 min at 94°C;  30 cycles of denaturation at 95°C for 30 sec, annealing at 60°C for 30 sec, and elongation at 72°C for 30 sec; final elongation for 5 min at 72°C | 742 |
| *virB* | forward: GGCTACATCGAAGATCGTATGAATG  reverse: GACTATAGCGATGGTTACGATGTTGAC | Initial denaturation for 4 min at 94°C;  35 cycles of denaturation at 94°C for 30 sec, annealing at 58°C for 30 sec, and elongation at 72°C for 30 sec; final elongation for 5 min at 72°C | 670 |
| *CYP11A1(2)* | forward: GCCACATCGAGAACTTCCAGAAG  reverse: CTGGTGTGGAACATCTTGTAGACG | Initial denaturation for 4 min at 94°C;  30 cycles of denaturation at 94°C for 60 sec, annealing at 64°C for 60 sec, and elongation at 72°C for 30 sec; final elongation for 5 min at 72°C | 520 |
| 25S-rRNA | forward: CGCTGTCTACGAGTCGGGTTGT  reverse: GGAGGGAACCAGCTACTAGACGGT | Initial denaturation for 4 min at 94°C;  30 cycles of denaturation at 94°C for 60 sec, annealing at 64°C for 60 sec, and elongation at 72°C for 30 sec;  final elongation for 5 min at 72°C | 709 |
