# Supplementary material for: A key enzyme of animal steroidogenesis can function in plants enhancing their immunity and accelerating the processes of growth and development
Source: BMC Plant Biol. 2017 Nov 14;17(Suppl 1):189. doi: 10.1186/s12870-017-1123-2 (PMC5688476; doi:10.1186/s12870-017-1123-2)
Supplement: Supplementary file 3 — Control (1, hight – 15 cm) and transgenic (2, hight - 72 cm) tobacco plant after one month of growth in the field conditions. (DOC 157 kb) [file 12870_2017_1123_MOESM3_ESM.doc]

**Additional File 3.**


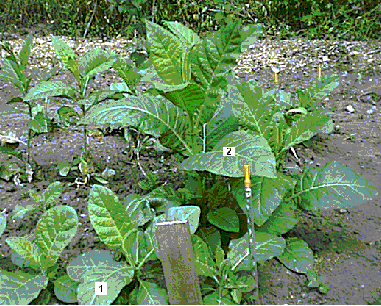


**Additional File 3.** Control (**1**, hight – 15 cm) and transgenic (**2**, hight - 72 cm) tobacco plant after one month of growth in the field conditions.
