# Supplementary material for: A key enzyme of animal steroidogenesis can function in plants enhancing their immunity and accelerating the processes of growth and development
Source: BMC Plant Biol. 2017 Nov 14;17(Suppl 1):189. doi: 10.1186/s12870-017-1123-2 (PMC5688476; doi:10.1186/s12870-017-1123-2)
Supplement: Supplementary file 4 — Assessment of fungicidal properties of transgenic tobacco plants. (DOC 28 kb) [file 12870_2017_1123_MOESM4_ESM.doc]

**Additional File 4.** Assessment of fungicidal properties of transgenic tobacco plants.

| Plant | Quantity of ungerminated spores, % | Quantity of germinated spores, % |
| --- | --- | --- |
| Control (wild type) | 10 ± 4 | 90 ± 6 |
| Control (with empty vector) | 15 ± 6 | 85 ± 8 |
| Transgenic (with *CYP11A1* cDNA) | 86 ± 12 | 14 ± 7 |
