# Supplementary material for: A key enzyme of animal steroidogenesis can function in plants enhancing their immunity and accelerating the processes of growth and development
Source: BMC Plant Biol. 2017 Nov 14;17(Suppl 1):189. doi: 10.1186/s12870-017-1123-2 (PMC5688476; doi:10.1186/s12870-017-1123-2)
Supplement: Supplementary file 5 — Compound racemes with large sepals and undeveloped pistils and stamens of the transgenic line No. 7. (DOC 237 kb) [file 12870_2017_1123_MOESM5_ESM.doc]

**Additional File 5.**


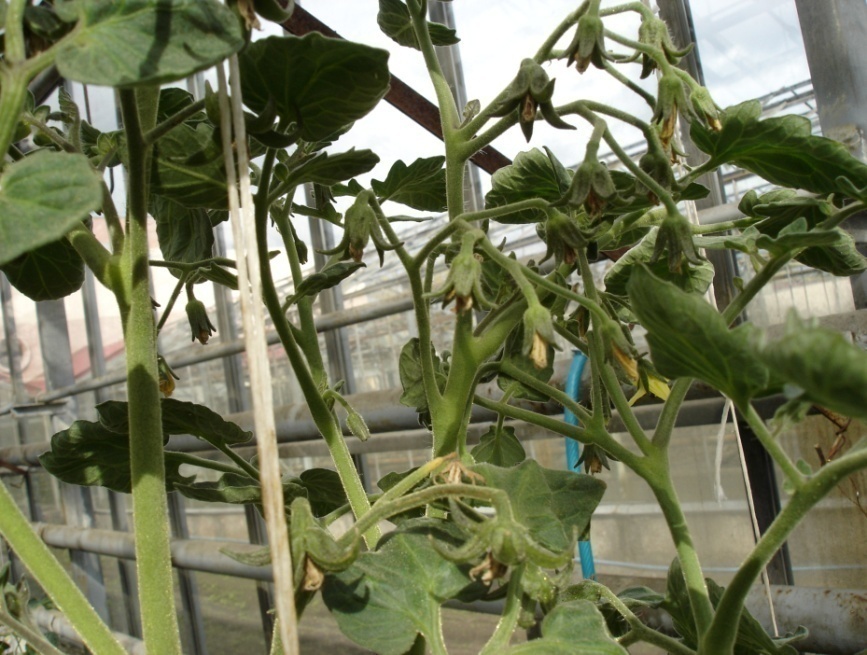


**Additional File 5.** Compound racemes with large sepals and undeveloped pistils and stamens of the transgenic line No. 7.
