# Supplementary material for: A key enzyme of animal steroidogenesis can function in plants enhancing their immunity and accelerating the processes of growth and development
Source: BMC Plant Biol. 2017 Nov 14;17(Suppl 1):189. doi: 10.1186/s12870-017-1123-2 (PMC5688476; doi:10.1186/s12870-017-1123-2)
Supplement: Supplementary file 6 — Fruit size and seed formation of the transgenic lines No. 4 and No. 7. (DOC 633 kb) [file 12870_2017_1123_MOESM6_ESM.doc]

**Additional File 6.**


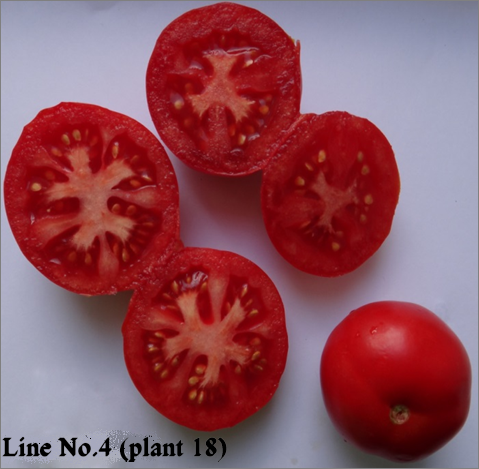

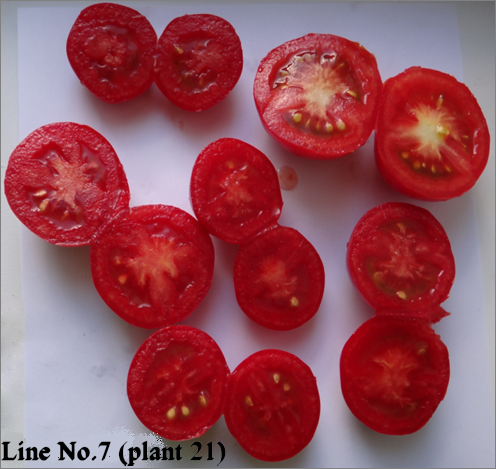


**Additional File 6.** Fruit size and seed formation of the transgenic lines No. 4 and No. 7.
