# Supplementary material for: A key enzyme of animal steroidogenesis can function in plants enhancing their immunity and accelerating the processes of growth and development
Source: BMC Plant Biol. 2017 Nov 14;17(Suppl 1):189. doi: 10.1186/s12870-017-1123-2 (PMC5688476; doi:10.1186/s12870-017-1123-2)
Supplement: Supplementary file 7 — Growth stages of the CYP11A1 transgenic tomato plants in the second decade of September. In the foreground – line No. 4 followed by the control variety Recordsmen (far behind), on the left side – line No. 7. Note the accelerated course of ontogenesis stages in the line No. 4 (as a result – precocity of some fruits and yellow leaves). Line No. 7 is characterized by greening and the intensive growth at the end of the vegetative season. (DOC 107 kb) [file 12870_2017_1123_MOESM7_ESM.doc]

**Additional File 7.**


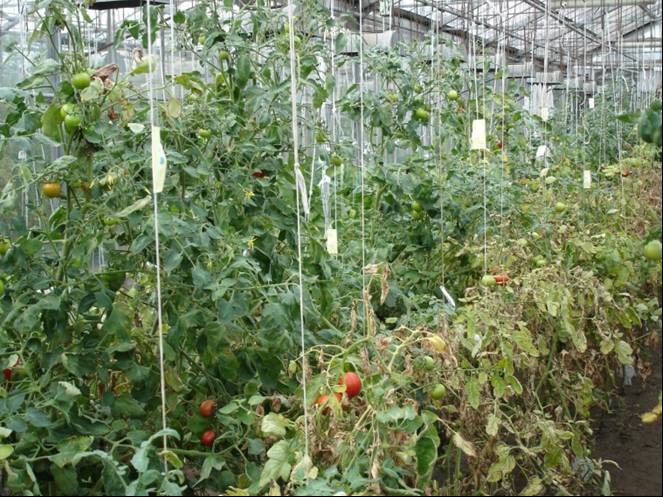


**Additional File 7.** Growth stages of the *CYP11A1* transgenic tomato plants in the second decade of September. In the foreground – line No. 4 followed by the control variety Recordsmen (far behind), on the left side – line No. 7. Note the accelerated course of ontogenesis stages in the line No. 4 (as a result – precocity of some fruits and yellow leaves). Line No. 7 is characterized by greening and the intensive growth at the end of the vegetative season.
