# Supplementary material for: A key enzyme of animal steroidogenesis can function in plants enhancing their immunity and accelerating the processes of growth and development
Source: BMC Plant Biol. 2017 Nov 14;17(Suppl 1):189. doi: 10.1186/s12870-017-1123-2 (PMC5688476; doi:10.1186/s12870-017-1123-2)
Supplement: Supplementary file 8 — Specific features of the CYP11A1 transgenic line No. 7: an intense green color of leaves and stem branches, large leaf blades with the corrugated surface, low amount of seeds in fruits, increased resistance to diseases. (DOC 72 kb) [file 12870_2017_1123_MOESM8_ESM.doc]

**Additional File 8.**


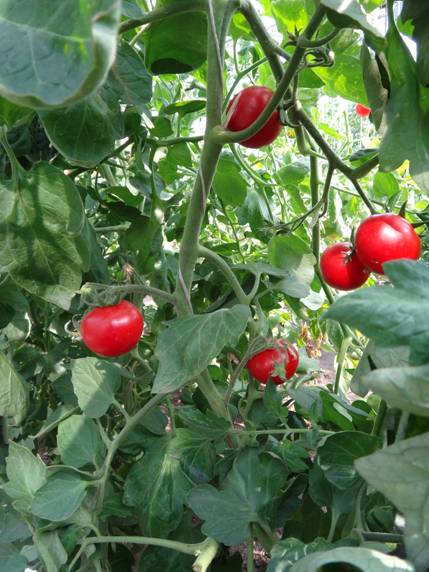


**Additional File 8.** Specific features of the *CYP11A1* transgenic line No. 7: an intense green color of leaves and stem branches, large leaf blades with the corrugated surface, low amount of seeds in fruits, increased resistance to diseases.
